# Signatures of disease progression in knee osteoarthritis: insights from an integrated multi-scale modeling approach, a proof of concept

**DOI:** 10.3389/fbioe.2023.1214693

**Published:** 2023-07-27

**Authors:** Ikram Mohout, Seyed Ali Elahi, Amir Esrafilian, Bryce A. Killen, Rami K. Korhonen, Sabine Verschueren, Ilse Jonkers

**Affiliations:** ^1^ Department of Movement Science, Human Movement Biomechanics Research Group, Leuven, Belgium; ^2^ Mechanical Engineering Department, Soft Tissue Biomechanics Group, Leuven, Belgium; ^3^ Department of Technical Physics, Biophysics of Bone and Cartilage Research Group, University of Eastern Finland, Kuopio, Finland; ^4^ Department of Rehabilitation Science, Research Group for Musculoskeletal Rehabilitation, Leuven, Belgium

**Keywords:** osteoarthritis, articular cartilage, finite element modeling, musculoskeletal modeling, adaptive modeling, collagen fibril degeneration, proteoglycans depletion, multiscale modeling

## Abstract

**Introduction:** Knee osteoarthritis (KOA) is characterized by articular cartilage degeneration. It has been widely accepted that the mechanical joint environment plays a significant role in the onset and progression of this disease. In silico models have been used to study the interplay between mechanical loading and cartilage degeneration, hereby relying mainly on two key mechanoregulatory factors indicative of collagen degradation and proteoglycans depletion. These factors are the strain in collagen fibril direction (SFD) and maximum shear strain (MSS) respectively.

**Methods:** In this study, a multi-scale in silico modeling approach was used based on a synergy between musculoskeletal and finite element modeling to evaluate the SFD and MSS. These strains were evaluated during gait based on subject-specific gait analysis data collected at baseline (before a 2-year follow-up) for a healthy and progressive early-stage KOA subject with similar demographics.

**Results:** The results show that both SFD and MSS factors allowed distinguishing between a healthy subject and a KOA subject, showing progression at 2 years follow-up, at the instance of peak contact force as well as during the stance phase of the gait cycle. At the peak of the stance phase, the SFD were found to be more elevated in the KOA patient with the median being 0.82% higher in the lateral and 0.4% higher in the medial compartment of the tibial cartilage compared to the healthy subject. Similarly, for the MSS, the median strains were found to be 3.6% higher in the lateral and 0.7% higher in the medial tibial compartment of the KOA patient compared to the healthy subject. Based on these intersubject SFD and MSS differences, we were additionally able to identify that the tibial compartment of the KOA subject at risk of progression.

**Conclusion/discussion:** We confirmed the mechanoregulatory factors as potential biomarkers to discriminate patients at risk of disease progression. Future studies should evaluate the sensitivity of the mechanoregulatory factors calculated based on this multi-scale modeling workflow in larger patient and control cohorts.

## 1 Introduction

Osteoarthritis (OA) is the most common joint disease, with the knee being the most frequently affected joint. This complex multi-factorial disease is characterized by degenerative changes in articular cartilage, subchondral bone and by synovial inflammation (Baliunas et al., 2002; Martel-Pelletier et al., 2016; Syed and Makris, 2020; Katz et al., 2021). Mechanical degeneration of articular cartilage is a hallmark of OA and a major contributor to joint dysfunction (Sinusas, 2012; Katz et al., 2021). Therefore, understanding biomechanical factors involved in cartilage degeneration is critical for developing effective therapeutic strategies to prevent or treat this debilitating disease.

Articular cartilage, an avascular connective tissue, is composed of specialized chondrocyte cells embedded in an extracellular matrix (ECM), which consists mainly of water, proteoglycans (PGs) and a collagen fiber network. The interactions between these constituents dictate the mechanical behavior of cartilage and enable it to distribute mechanical load across the joint surface (Sophia Fox et al., 2009; Heinegård and Saxne, 2011; Mukherjee et al., 2020). However, changes in mechanical loading during locomotion will alter stresses and strains within the tissue, which—upon exceeding a critical threshold—is associated with irreversible changes to the collagen network and depletion of the PG content (Speirs et al., 2014; Tanska et al., 2018; Elahi et al., 2021; Elahi et al., 2023). Indeed, the mechanical joint environment is considered to be a significant contributor to the disease process, with pathomechanical loading potentially leading to maladaptive responses of chondrocytes and ECM, as well as increased inflammation, further aggravating cartilage tissue degeneration (Hurwitz et al., 2000; Baliunas et al., 2002; Sun, 2010).

To non-invasively study the mechanical joint environment, *in silico* models have been developed. In particular, musculoskeletal (MSK) models have proven useful in estimating knee joint loading during locomotion, which has been found to be altered in patients with OA (Lloyd and Besier, 2003; Lenhart et al., 2015; Meireles et al., 2017). By combining MSK models with finite element (FE) models of the articular cartilage, it is possible to simulate the effects of mechanical joint loading on cartilage strain distributions (Adouni and Shirazi-Adl, 2014; Halonen et al., 2014; Halonen et al., 2017; Erdemir et al., 2019; Esrafilian et al., 2020; Esrafilian et al., 2021). In addition, articular cartilage FE models can be further enhanced by assigning complex material models that mimic the main constituents of cartilage ECM, allowing an even more detailed evaluation of cartilage tissue responses to mechanical loading (Wilson et al., 2005). Such *in silico* models provide a valuable platform for investigating the complex interplay between mechanical loading, cartilage structure and function, and constituent damage and depletion.

Cartilage degeneration algorithms, also referred to as adaptive algorithms have emerged that mimic time-dependent, OA-associated cartilage degeneration processes (Mononen et al., 2018; Orozco et al., 2018; 2021; Eskelinen et al., 2019; Elahi et al., 2021). The Cartilage Adaptive Reorientation Degeneration (CARED) model, namely, the most recently developed integrated adaptive model, uses a fibril-reinforced poroviscoelastic material model to calculate the strain in collagen fibrils and maximum shear strain as mechanoregulatory parameters that drive collagen degradation and PG depletion, respectively (Elahi et al., 2021; Elahi et al., 2023). Although the CARED model is able to predict degenerative changes in an explant model (osteochondral plug with 3 mm diameter) under different loading conditions, this is not representative for cartilage degeneration in the whole knee joint. To extend the insights on the impact of mechanical loading to cartilage degeneration *in vivo*, we need to define a multi-scale modeling workflow that includes a whole joint to tissue analysis and that relies on a complex cartilage material model to allow evaluating the impact of the mechanical tissue environment on constituent degeneration.

Whole knee joint FE models in combination with adaptive models have been developed (Mononen et al., 2018; Orozco et al., 2021) to predict collagen fibril degradation in the native knee joint (Mononen et al., 2016) and, more recently, to predict proteoglycan depletion and collagen fibril re-orientation (Heck et al., 2015; Orozco et al., 2018). To the best of our knowledge such algorithms have not been used to study cartilage degenerative processes specifically in early-stage OA cohorts. As such, insight into mechanoregulatory factors that predispose to cartilage degeneration in early disease stages remain undocumented.

This study proposes a unique, multi-scale, patient-specific *in silico* approach to evaluate the mechanical environment of the human knee joint based on loading conditions derived from MSK modeling during gait in a healthy and an early-stage knee OA (KOA) subject displaying (fast) progression over a 2-year time frame. These subject-specific loading and boundary conditions were estimated based on experimental gait analysis data collected at baseline, prior to a 2-year follow-up, which is processed through a MSK modeling workflow. The proposed modeling pipeline in this study uses a unique MSK modeling-informed approach, where the subject-specific boundary and loading conditions are used to drive an FE model that is then used to evaluate the mechanical environment of cartilage and meniscus tissues within the knee joint. Firstly, mechanoregulatory factors known to underly cartilage constituent damage were evaluated, at baseline, to characterize the mechanical tissue environment prone to cartilage degeneration. More specifically, strain in fibril direction (SFD) and maximum shear strain (MSS) in the articular cartilage were evaluated as mechanoregulatory factors driving collagen degradation and PG depletion, respectively. Secondly, tissue responses in the menisci, a structure known to participate in strain mitigation and protection of the underlying cartilage tissue (Englund, 2009; Netravali et al., 2010; Choi et al., 2014; Verdonk et al., 2016), were analyzed to evaluate their role in altering the mechanical environment of the cartilage tissue in the presence of subject-specific loading conditions. We hypothesize that under subject-specific loading conditions and consistent use of generic knee articular geometries in both MSK and FE modeling workflows, the mechanoregulatory factors are distinct between a healthy and a KOA subject and that the menisci are differentially implicated in load transmission to the cartilage surface. If confirmed, we anticipate that this study will provide proof of concept data showing the potential of these mechanical factors as functional biomarkers to discriminate patients at risk of (fast) disease progression.

## 2 Methods

**FIGURE 1:**
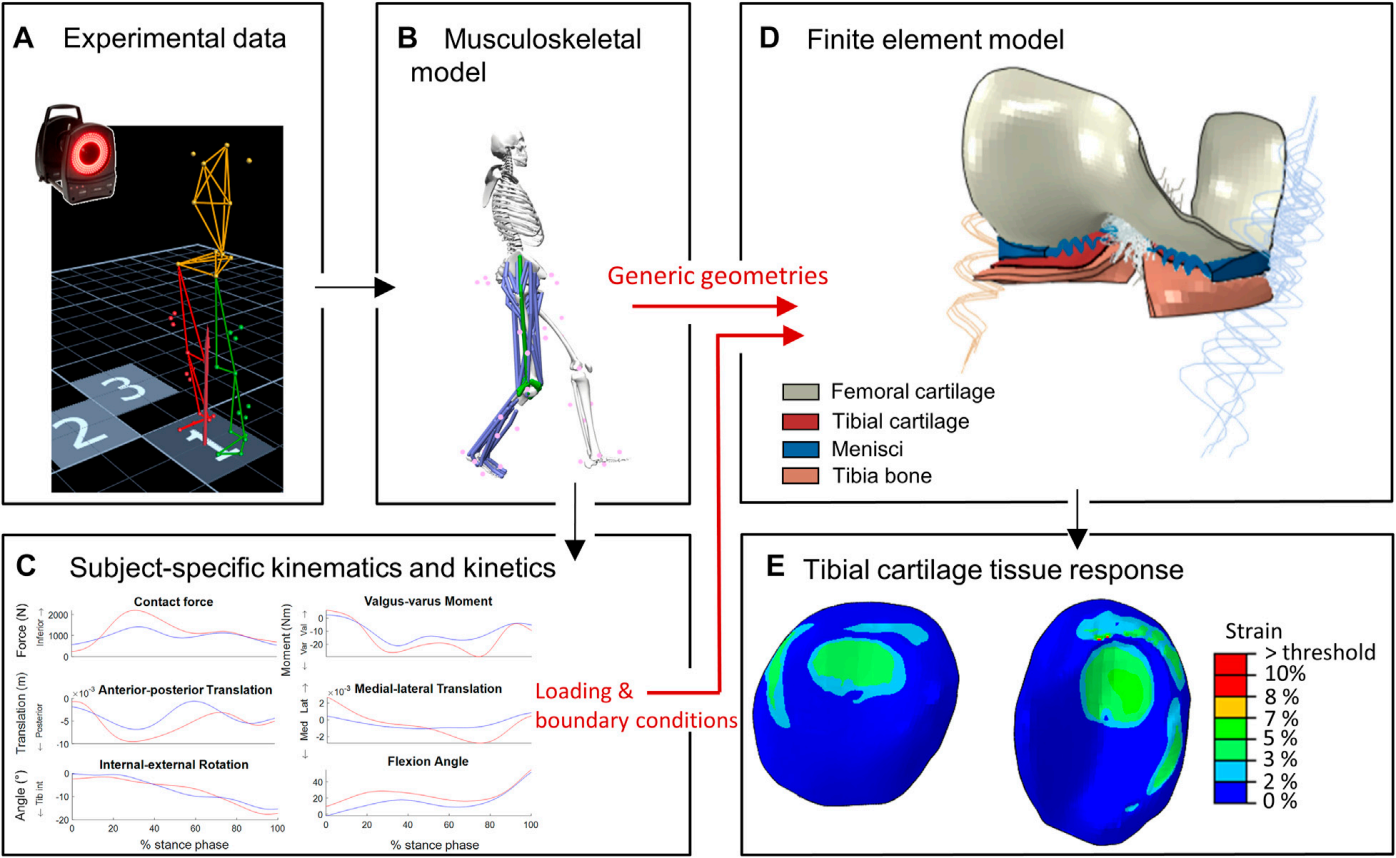
Workflow of the study: **(A–C)** represent the MSK modeling pipeline to estimate subject-specific knee joint kinematics and kinetics **(D,E)** represent the FE modeling pipeline to estimate cartilage tissue responses. Red arrows indicate unique approaches: consistent knee geometries in MS and FE pipelines, and MSK-workflow derived subject-specific loading and boundary conditions as inputs for FE model.

The study workflow, illustrated in Figure 1, consisted of consecutive MSK and FE modeling pipelines. The red arrows indicate two unique methodological features of this study: 1) consistency in the use of the knee articular geometries in both MSK and FE modeling pipelines and 2) MSK modeling-informed subject-specific loading and boundary conditions as inputs to the developed FE model.

One healthy control subject and one progressing early KOA subject with similar demographics (Table 1) were selected from a longitudinal historical dataset (Baert et al., 2013; Mahmoudian et al., 2016). The selection of the KOA subjects was based on the classification criteria of Luyten et al. (2012): More specific, a subject with a minimum increase of 1 Kellgren-Lawrence (KL) score (Kellgren and Lawrence, 1957) - evaluated by the same rater blinded for disease or follow-up status - over a two-year follow-up. The patient had no history of prior trauma or surgery with regards to the lower limbs and back. Furthermore, the patient did not have any musculoskeletal disorders (other than KOA) nor did they suffer from any neurological diseases that would affect their balance or coordination during gait during a six-months period preceding the data collection. All procedures were approved by the local ethical committee (S50534) and written informed consent was obtained from the participants.

**TABLE 1:** Patient demographics *Kellgren-Lawrence classification score for a right knee lateral compartment progressor at baseline and after a 2-year follow-up.

Subject type	Gender	Age (years)	Weight (kg)	Height (m)	Body mass index (BMI) (kg/m^2^)	KL-score baseline*		KL-score follow-up*	
						medial	lateral	medial	lateral
Control	Female	72	61	1.56	25	0	0	0	0
KOA	Female	70	68	1.56	28	1	1	1	2

### 2.1 MS-based estimation of patient-specific knee joint kinematics and kinetics

#### 2.1.1 Experimental data collection

Experimental gait analysis data from the aforementioned longitudinal historical dataset (Baert et al., 2013; Mahmoudian et al., 2016; Meireles et al., 2017) was analyzed at baseline (before the two-year follow-up). This included three-dimensional marker position data acquired with a motion capture system (10MX Vicon, 100 Hz) and ground reaction force data collected with in-ground force plates (AMTI, 1000 Hz). First, a static calibration trial was performed (Cappozzo et al., 1995), followed by four trials of over-ground walking at participant’s self-selected speed. The data were then converted using a custom Matlab script (MATLAB R2020b, The Math Works, Inc., Natick, Massachusetts, United States ) as a pre-processing step for the MSK workflow.

#### 2.1.2 Musculoskeletal modeling

The OpenSim Joint Articular Mechanics (JAM) (Smith et al., 2014) modeling workflow was used to estimate knee joint kinematics, moments and loading. A 3D MSK model with 6 degrees of freedom (DoF) tibiofemoral (TF) and patellofemoral joint kinematics (Lenhart et al., 2015) was used and included the major knee ligaments which were modeled as non-linear spring bundles (Blankevoort and Huiskes, 1991). The state-of-the-art OpenSim-JAM workflow uses standard OpenSim tools (Delp et al., 2007) in combination with a unique knee joint contact model capable of estimating joint secondary coordinates (Anterior-posterior and medial-lateral translations, internal-external and adduction-abduction rotation). As such, the menisci are not explicitly modeled but are included in the parameters of the knee contact model.

The experimental gait analysis data is processed through the MSK OpenSim-JAM workflow. First, the generic model is scaled to the subject’s anthropometry, based on experimental marker data from the static trails. Then an inverse kinematics algorithm was used to estimate joint angles (i.e., primary kinematics) based on a minimization of marker position between the model and experimental data (Lu and O’Connor, 1999). The concurrent optimization of muscle activations and kinematics (COMAK) algorithm (Smith et al., 2018), as part of OpenSim-JAM, was used to simulate the desired secondary tibiofemoral kinematics and contact forces.

The following specific outputs of the OpenSim-JAM were used as boundary conditions for the FE model: TF anterior-posterior translation, TF medial-lateral translation, TF internal-external rotation, knee flexion angle, varus-valgus moment and superior-inferior knee joint contact force. One representative trial for each subject was processed, more details on the outcome of the MS simulations are presented in Supplementary Material.

### 2.2 Finite element modeling of cartilage tissue responses

#### 2.2.1 Geometries and mesh

FE models of the human knee joint were created using Abaqus software (Dassault Systèmes, United States), that includes the tibial and femoral cartilage surfaces, (part of the) tibia bone and the menisci based on previous FE models developed by Esrafilian et al. (2020), Halonen et al. (2016) and Bolcos et al. (2018) which were validated against experimental data (Halonen et al., 2017; Esrafilian et al., 2020; Esrafilian, 2021; Esrafilian et al., 2022). The patella was not included in this model as this study focusses on the tibiofemoral cartilage tissue responses.

**FIGURE 2:**
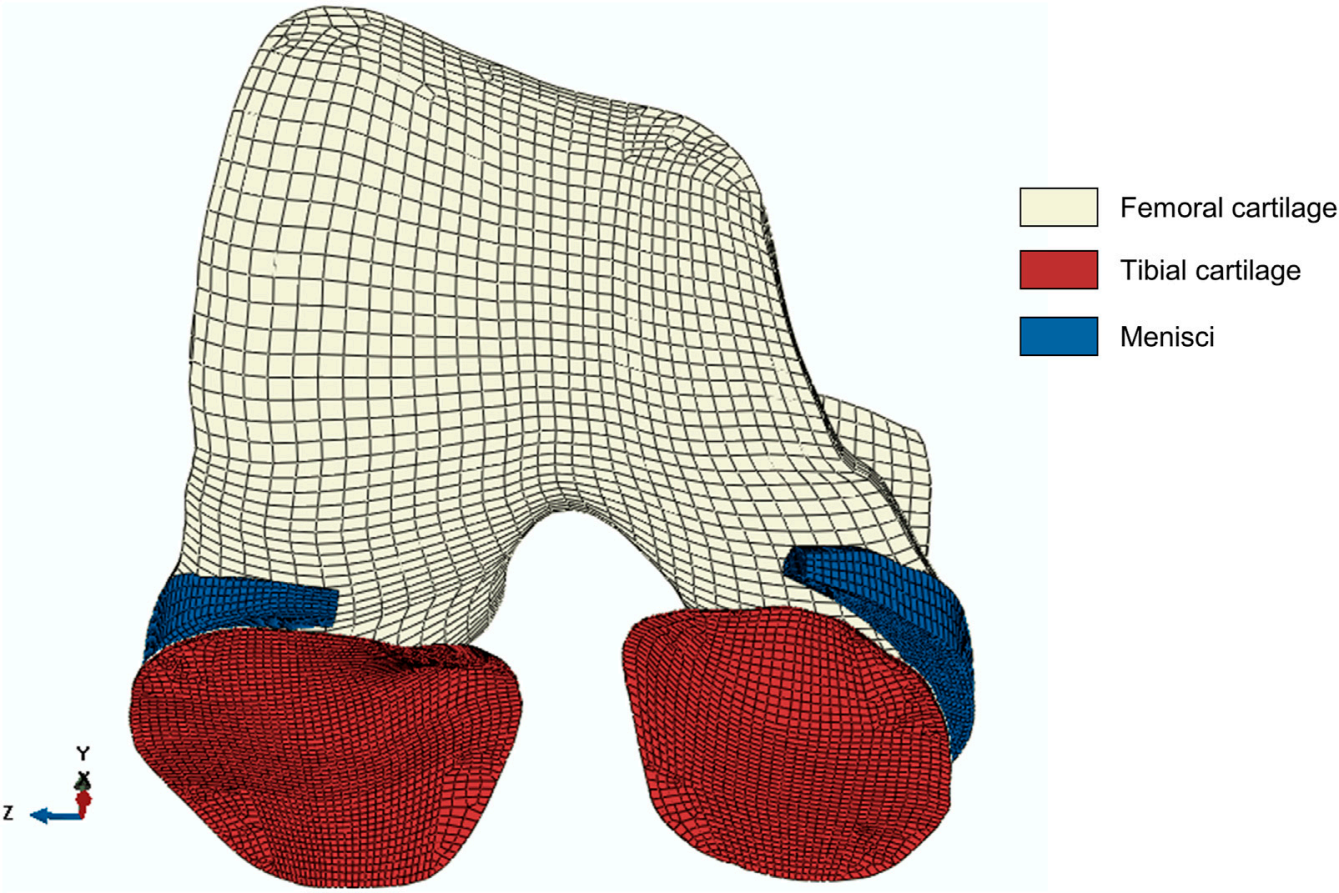
The hexahedral mesh of the soft tissues in the model: tibial cartilage (33,250 elements), femoral cartilage (8,880 elements) and menisci (7,680 elements). All soft tissue parts consist of four layers. The tibial cartilage mesh consists of two finer superficial layers and two coarse bottom layers to facilitate convergence (Halonen et al., 2016). The x-, y-, z-directions represent the anterior-posterior, superior-inferior and medial-lateral direction, respectively.

Identical generic knee joint geometries were used in the FE model and the MSK knee contact model to enforce geometrical consistency between the two workflows. Hexahedral meshes, generated in ANSA (v21.0.1, BETA CAE Systems International AG, Switzerland), were based on the meshes of the Lenhart et al. (2015) knee contact model that were derived from MRI segmentations of a healthy subject. Given that the KOA patient presented with KL score 1 indicative of early OA, no structural changes in the cartilage such as cartilage thinning were assumed to be present. Hence the use of a generic mesh was deemed acceptable. Despite not being incorporated in the MSK knee contact model, the designated mesh of the menisci was taken from the same healthy subject and was used to generate the hexahedral mesh for the FE model. Soft tissues (apart from the ligaments) were modeled as porous 8-node elements were used (C3D8P element type), with a total of 49,810 elements (femoral cartilage = 8,880 elements, menisci = 7,680 elements, tibial cartilage = 33,250 elements). Figure 2 shows the hexahedral meshes of the tibial cartilage, femoral cartilage and menisci. The Tibia bone was modeled as 675 8-node elements (C3D8 element type). A mesh convergence test was previously performed by Halonen et al. (2016) to verify the mesh size and number of elements in the model. To further enhance the model convergence, the superficial element layers of tibial cartilage have been subdivided resulting in a four-layered mesh with two finer superficial layers and two more coarse bottom layers as suggested by Halonen et al. (2016). Furthermore, the FE model included the anterior cruciate ligament (ACL), posterior cruciate ligament (PCL), lateral collateral ligament (LCL) and medial collateral ligament (MCL), using identical insertion points, origins and stiffness as in the MSK model. The ligaments were modeled as non-linear springs (SpringA element type, (Blankevoort and Huiskes, 1991)).

#### 2.2.2 Material model

Cartilage was modeled as a fibril-reinforced poroviscoelastic (FRPVE) material including a biphasic (fluid and solid phases) description with a hyperelastic Neo-Hookean solid matrix representing the PGs and a viscoelastic fibril network representing collagen fibrils (Wilson et al., 2004; 2005; Julkunen et al., 2007). The primary collagen fibrils followed the depth-dependent Benninghoff-type arcade architecture and the superficial collagen orientation was indicated by split lines towards the center of area (See Supplementary Figure S2 in the Supplementary Material). Furthermore, the fibril density and the fluid fraction also varied throughout the depth of the tissue (M. E. Mononen et al., 2012; Esrafilian, et al., 2020; Benninghoff, 1925; Goodwin et al., 2004). The effect of these depth-wise material properties on cartilage tissue stresses and strains have been analyzed in a previous study by Halonen et al. (2013). For the menisci, a fibril-reinforced poroelastic (FRPE) material was used, which is analogous to the FRPVE material model apart from the collagen fibrils which were modeled as an elastic fibril network. A linear elastic material model was used for tibia bone and the material parameters of the ligaments were taken from the MSK model (Blankevoort and Huiskes, 1991; Currey, 2004; Lenhart et al., 2015). A more detailed description of the material model and specific material parameters (Wilson et al., 2004; Julkunen et al., 2007; Makris et al., 2011; Dabiri and Li, 2013) is provided in the Supplementary Material. The same material model and material parameters were used for both subjects. Considering the KOA patient’s KL score of 1, indicating early OA, this assumption was considered acceptable.

#### 2.2.3 Subject-specific loading and boundary conditions

The time-dependent loading and boundary conditions derived from the MSK workflow were applied throughout the stance phase of the gait cycle. This was done by means of a transient analysis, more specifically a soils consolidation analysis, with an implicit FE scheme. The following subject-specific loading and boundary conditions derived from the MS model were implemented at the reference point of the femur in the FE model: Anterior-posterior translation, medial-lateral translation, internal-external rotation, knee flexion angle, varus-valgus moment and the superior-inferior TF joint contact force. This reference point was located between the medial and lateral femoral epicondyles and was tied to the femoral cartilage-bone interface. The origins of the ligaments were also tied to this reference point. The FE model was driven at this femoral reference point and thus the output of the MS model was extracted from the local coordinate system of the proximal tibia as this corresponds to the relative motion of the femur to the tibia.

Femur bone was considered rigid and its effect was included in the FE model with encaster boundary condition on the femoral cartilage internal surface. The tibia bone was modeled as linear elastic for the purpose of facilitating the convergence of the model to a numerical solution. The bottom surface of the tibial bone was encastred. The following frictionless contact types were defined in the model as nodes to surfaces: Tibial cartilage to femoral cartilage, menisci to femoral cartilage, tibial cartilage to menisci and menisci to tibia bone. The assumption of frictionless contact is widely accepted as the standard in knee joint FE modeling, primarily because the main function of articular cartilage is to provide a smooth and frictionless surface for joint articulation and efficient load distribution across the joint surface (Mukherjee et al., 2020).

### 2.3 Analysis of FE model results

#### 2.3.1 Evaluation of cartilage tissue response

This study analyzed maximum shear strain (MSS, see Eq. 1) and strain in fibril direction (SFD, see Eq. 2). Thresholds of MSS = 30% and SFD = 10% were chosen as being indicative of PG depletion and collagen degeneration respectively, as implemented in the most recently developed CARED model (Elahi et al., 2021) and based on literature findings (Eskelinen et al., 2019).

$$\varepsilon_{\max}=\max\;\left\{\left|{ɛ}_{p,1}-{ɛ}_{p,2}\right|,\left|{ɛ}_{p,1}-{ɛ}_{p,3}\right|,\left|{ɛ}_{p,2}-{ɛ}_{p,3}\right|\right\},$$ (1)

where ɛ_p,1_, ɛ_p,2_, ɛ_p,3_ are the principal strains of the Green-Lagrangian strain tensor.

$$\varepsilon_{f}=\ln\;\left(\left\|Fe_{f}\right\|\right),$$ (2)

where **F** is the deformation gradient tensor and **e_f_** is the unit vector of the fibril orientation.

First, tibial cartilage tissue responses were evaluated throughout the entire stance phase of the gait cycle. Here, the analysis was restricted to the tibial cartilage volume related to the contact area, this area amounts to 20% of the total tibial cartilage volume as observed in the model, as tissue strains (mostly) outside the contact area between femoral and tibial cartilage and between menisci and tibial cartilage were considerably low and did not contribute to the analysis. The strains were extracted from the centroids of the tibial cartilage elements and element volumes were taken into account. Then, tibial cartilage tissue responses were evaluated at peak superior-inferior knee contact force, as tissue strains reached their absolute maxima at this time-point during the stance phase. Results were evaluated qualitatively based on strain responses at the tibial cartilage surface and quantitatively by means of violin plots of the tissue strains within the cartilage volume. This quantitative analysis was also restricted to the tibial cartilage volume related to the contact area.

#### 2.3.2 Evaluation of meniscal tissue response

The maximum principal strain (MPS) of the logarithmic strain tensor was analyzed to reflect the overall tissue response of the menisci. The MPS was not directly associated with structural changes in the menisci. The tissue response was evaluated at peak superior-inferior knee contact force to be consistent with the evaluation of the tibial cartilage. Analogous to the evaluation of the tibial cartilage, a qualitative evaluation was done based on the strain at the (superior) surface of the menisci and a quantitative analysis was performed by means of violin plots of the tissue responses within the menisci. Here, the whole volume of the menisci was included in the quantitative analysis as (nearly) the whole surface of the menisci was in contact with either the femoral or tibial cartilage.

## 3 Results

### 3.1 Evaluation of the cartilage tissue response

#### 3.1.1 Strain evolution throughout the stance phase of the gait cycle

**FIGURE 3:**
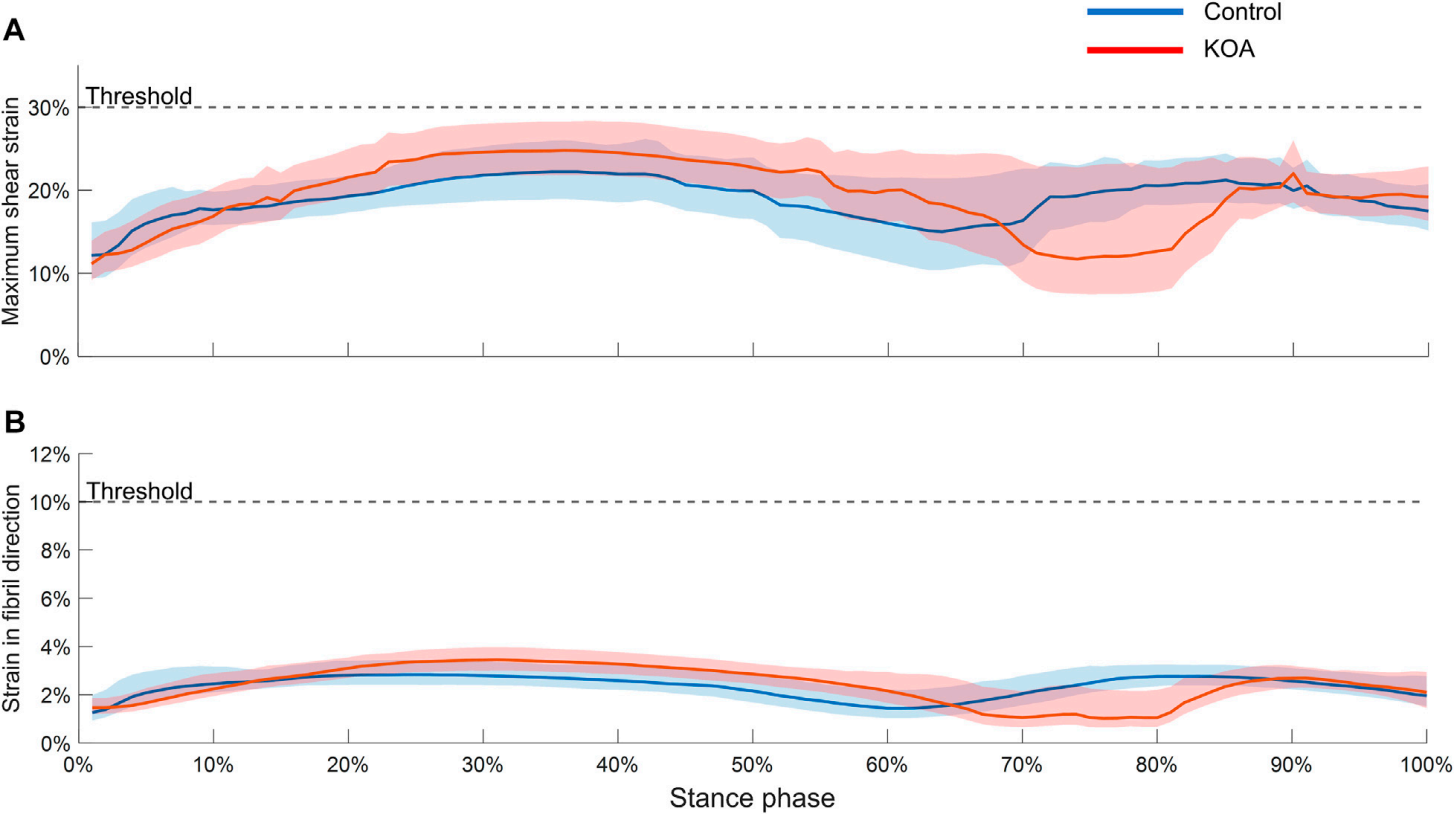
Maximum shear strain **(A)** and strain in fibril direction **(B)** evaluated throughout the stance phase in both tibial compartment volumes. The graphs represent the median strain and the lower and upper boundaries of the shaded areas represent the 25th and 75th percentile, respectively.

During the majority of the stance phase, the MSS (Figure 3A) and SFD (Figure 3B) were higher in the KOA subject than in the control subject. However, neither the median nor the 75th percentile boundary exceeded the degeneration thresholds of MSS and SFD.

#### 3.1.2 Strain in fibril direction at peak contact force

**FIGURE 4:**
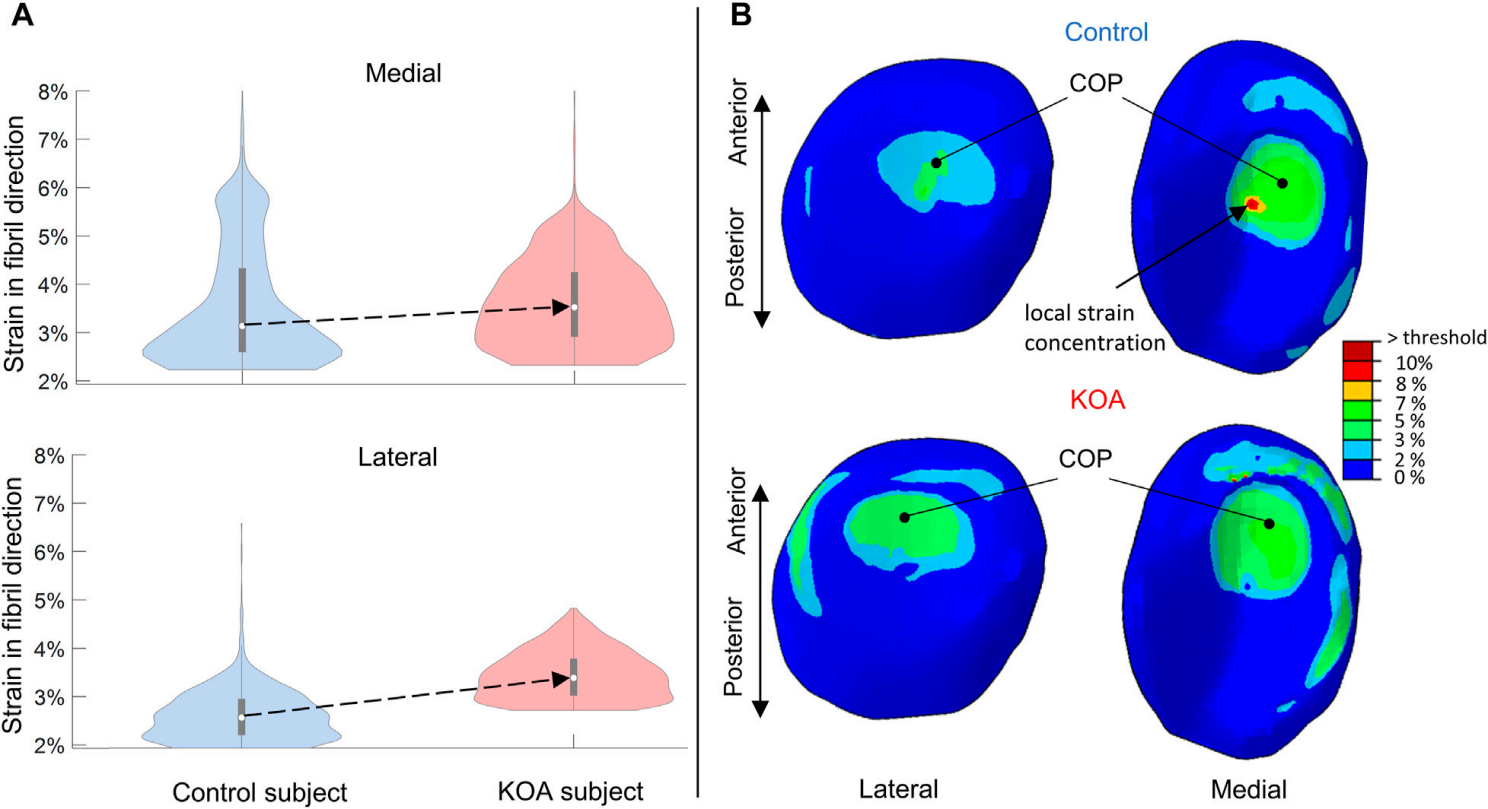
Strains in fibril direction (SFD) evaluated at peak contact force: **(A)** Violin plot (depicting the 25th percentile (first quartile), median (second quartile), 75th percentile (third quartile), density trace (smoothed histogram) and whiskers (based on 1.5 interquartile range value)) of the SFD (%) of the medial (top) and lateral (bottom) volumes of the tibial compartment for the control (blue) and KOA (red) subject. **(B)** SFD (%) in the lateral (left) and medial (right) tibial cartilage of the control (top) and progressor (bottom) subjects. The local strain concentration in the control subject is indicated **(B)** which corresponds to the bulge at the upper tale of the violin plot **(A)** of the medial compartment in the control subject. COP indicates the center-of-pressure in each tibial compartment.

For both lateral and medial compartments, the strain in fibril direction (SFD) was more elevated in the KOA compared to the control subject, with the median being 0.82% higher in the lateral and 0.4% higher in the medial compartment respectively (Figure 4A). With the exception of some local strain concentrations, overall, the SFD thus did not exceed the threshold of collagen fibril degeneration (10%). Figure 4B shows a posterior shift in center-of-pressure (COP) in the KOA subject of 2.9 mm in the lateral compartment and 5.1 mm in the medial compartment compared to the control subject.

#### 3.1.3 Maximum shear strain at peak contact force

**FIGURE 5:**
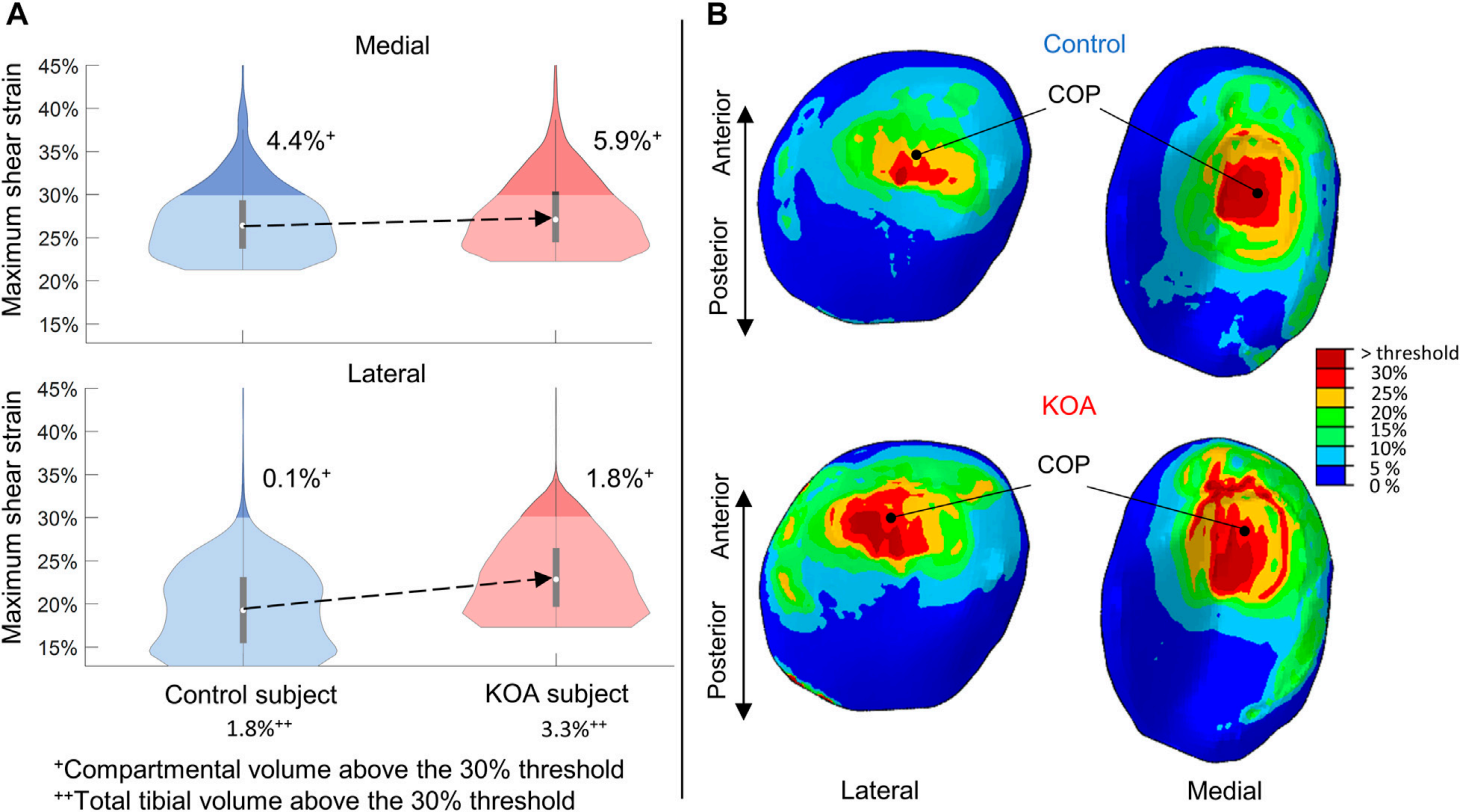
Maximum shear strain (MSS) evaluated at peak contact force: **(A)** Violin plot (depicting the 25th percentile (first quartile), median (second quartile), 75th percentile (third quartile), density trace (smoothed histogram) and whiskers (based on 1.5 interquartile range value)) of the MSS (%) of the medial (top) and lateral (bottom) volumes of the tibial compartment for the control (blue) and KOA (red) subject. **(B)** MSS (%) in the lateral (left) and medial (right) tibial cartilage of the control (top) and progressor (bottom) subject. COP indicates the center-of-pressure in each tibial compartment.

For both compartments, the MSS of the KOA subject was more elevated compared to the control subject (Figure 5), with the median being 3.6% higher in the lateral tibial compartment and 0.7% in the medial tibial compartment respectively (Figure 5A). The MSS did surpass the threshold of degeneration (above 30%) and a larger volume was affected in the KOA subject (Figure 5A).

### 3.2 Evaluation of the meniscal tissue response: maximum principal strain at peak contact force

**FIGURE 6:**
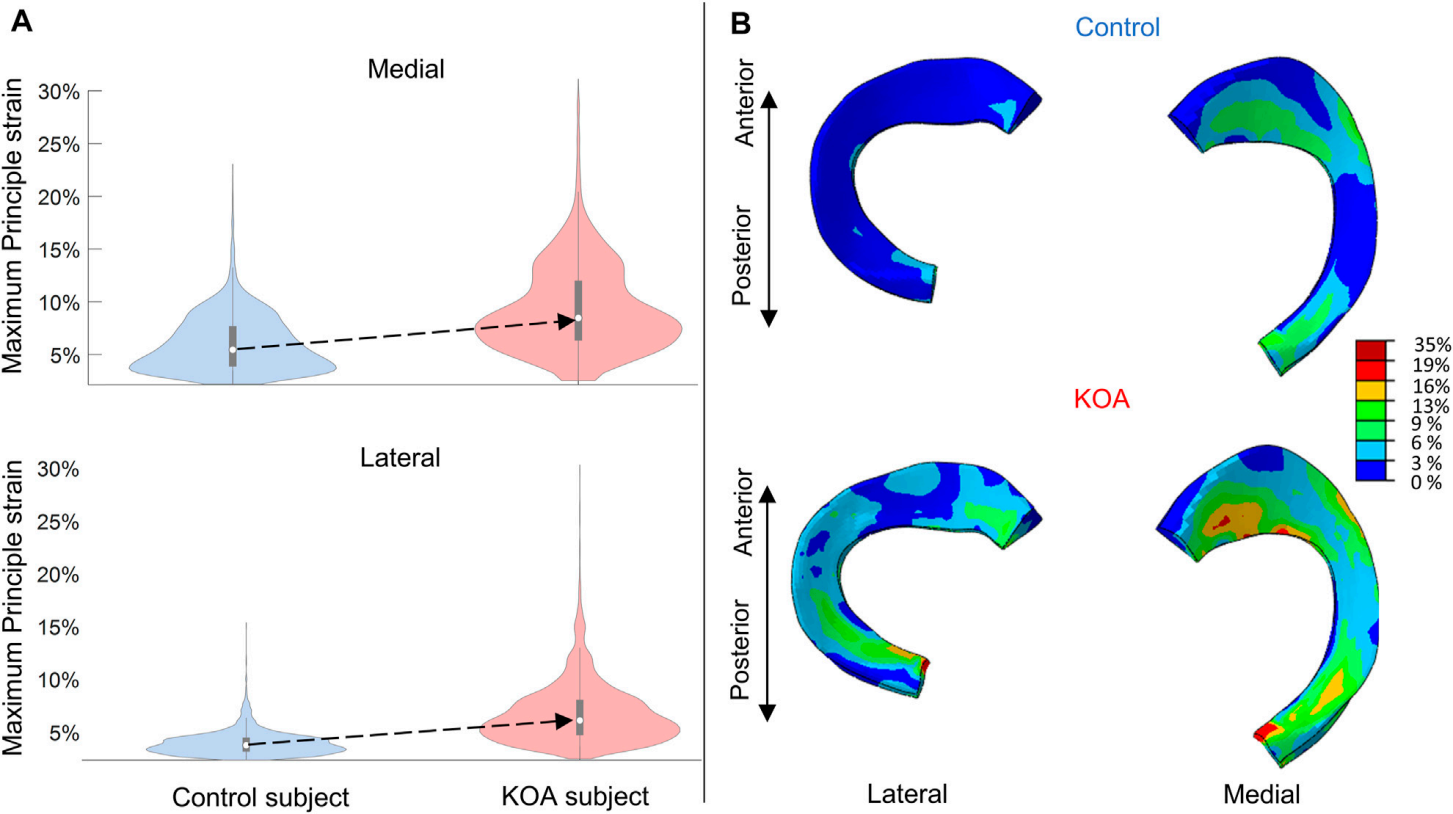
Maximum principal strain (MPS) evaluated at peak contact force: **(A)** Violin plot (depicting the 25th percentile (first quartile), median (second quartile), 75th percentile (third quartile), density trace (smoothed histogram) and whiskers (based on 1.5 interquartile range value)) of the MPS (%) of the medial (top) and lateral (bottom) menisci volumes for the control (blue) and KOA (red) subject. **(B)** MPS (%) in the lateral (left) and medial (right) menisci of the control (top) and progressor (bottom) subject.

The maximum principal strain (MPS) is more elevated in the KOA subject compared to the control subject (Figure 6) The median MPS of the KOA subject is 2.5% and 3.1% higher for lateral and medial meniscus compared to the control, respectively.

## 4 Discussion

This study is the first to compare key mechanoregulatory factors associated with cartilage tissue degeneration between a control subject and an early-stage KOA patient (KL 1) showing disease progression in 2 years. The study focused on two mechanoregulatory factors, namely, strain in the fibril direction (SFD) and maximum shear strain (MSS) and evaluation was performed using subject-specific loading conditions determined during the baseline stance phase of gait.

Using the proposed multi-scale patient-specific *in silico* approach, the local cartilage strains in terms of SFD and MSS, were found to be higher for the KOA patient compared to the control subject throughout the majority of stance duration and at peak contact force. This shows how these intra-tissue strains during locomotion are distinct between the healthy and early KOA subject already at baseline despite the subjects’ similar demographics. During stance, this distinction is even more clear when evaluating the 75th percentile of the tissue strains that were elevated throughout the gait cycle in the OA subject. This is in line with previous suggestions that not only instantaneous loading that surpasses the threshold of cartilage degeneration, but also the accumulated load over time (cumulative load) during normal ambulatory activities, could induce cartilage degeneration (Mononen et al., 2016). Furthermore, the strain difference at peak contact force between subjects is most prominent in the lateral compartment where after 2 years follow-up, structural progression can be observed in the KOA subject. This could invigorate our hypotheses that the mechanoregulatory factors studied here may serve as functional biomarkers with the potential to discriminate patients at risk of disease progression already in the early phases of the disease process. Indeed, the KOA subject presented with structural lateral compartment OA progression after two-year follow-up as evidenced by an increase in KL score (KL score 1 at baseline to KL score 2 after two-year follow-up).

Importantly, not only the mechanical environment of the cartilage surface is altered, also the maximum principal strain in the menisci is consistently higher in the KOA compared to the control subject, which is likely also due to the posterior shift in the center-of-pressure. This suggests an important role for the meniscus in strain mitigation in early KOA subjects. Any deficiency in meniscal integrity could therefore even further amplify the excessive articular cartilage loading. This is in line with literature where (partial) meniscectomy and meniscal subluxation have been associated with the degeneration process of cartilage (Choi et al., 2014; Verdonk et al., 2016). It is important to note that only one KOA subject was included in this proof-of-concept study. Hence, these findings will need to be validated in larger cohorts.

The altered joint level loading during gait in the patient with KOA impacts the local cartilage micro-environment such that constituent depletion occurs. Indeed, although the SFD was well below the threshold of degeneration (10%), MSS surpassed the threshold (30%) in 3.3% of the cartilage volume of the KOA subject. This would suggest a potential risk of PG depletion to drive the OA progression rather than collagen degeneration, which is highly relevant as it has been suggested that damaged cartilage can suffer from PG depletion under physiological loading conditions (Orozco et al., 2018) and that these changes in PG content can still be reversible which is thought not to be the case for changes in the collagen network (Elahi et al., 2023). An unexpected finding of this study was that 1.8% of the tibial cartilage volume of the control subject also surpassed the MSS threshold of degeneration, suggesting PG depletion. This could be attributed to age or possibly computational limitations as local strain concentrations in the models were found specifically when the load-bearing contact location was at the center of the respective tibial compartments. This is more prominent in the control subject as a posterior shift in contact location was found in the KOA subject.

Finally, in this study, personalized loading conditions were estimated from MSK modeling and used to drive the FE model to study the tissue strain responses. These personalized loading conditions estimated during gait differed between the control subject and the KOA patient, with higher inferior-superior contact force and knee adduction moment, as well as higher knee flexion in the KOA compared to the control subject. Previous studies have shown that the aforementioned characteristics are distinctive for KOA subjects (Hurwitz et al., 2000; Kaufman et al., 2001; Al-Zahrani and Bakheit, 2002; Baliunas et al., 2002; Astephen et al., 2008). Additionally, the posterior shift in center-of-pressure is in-line with previous studies that reported a posterior-lateral shift in center-of-pressure and load-bearing area when comparing KOA subjects to control subjects (Andriacchi and Mündermann, 2006; Meireles et al., 2017). These findings highlight the need to use disease-specific kinematic-kinetic input to the whole knee FE and adaptive workflow modeling, for instance using integrated 3D gait analysis data is used within an integrated MSK and FE modeling workflow to fully capture the altered mechanical environment in the cartilage tissue in the homeostatic joint and a joint susceptible for OA disease progression.

### 4.1 Limitations and future work

In this study, proof of concept data was obtained from two participants which could serve as a stepping stone for future analysis where more participants are included. It would be particularly interesting to include non-progressive KOA subjects as this would provide more information and likely more insight into the relation between localized tissue strains and cartilage degeneration. It must be noted that executing the FE workflow is a time-consuming task with high computational costs, and it can be challenging to achieve convergence of the model. The advantage of the implemented modeling approach is that it uses generic geometries consistent with the MSK pipeline. This alleviates the need for re-meshing of the geometries, which saves a significant amount of time, however at the expense of losing personalized details on the articular cartilage geometry. Thus, variations in cartilage thickness are not accounted for. Although the use of a generic mesh was deemed acceptable in this study, as no cartilage thinning was assumed to be present in the KOA patient with KL score 1, personalized geometry, in particular thickness, can further enhance the accuracy of strain predictions within the cartilages and menisci.

Although subject-specific loading conditions were applied, further personalization of the model in terms of cartilage thickness and geometry, knee contact with friction or tissue material properties in the FRPVE material model based on the disease state of the subject (healthy, early OA, late-stage OA) needs to be considered and might affect the SFD and MSS measures. Although state-of-the-art, the use of similar material parameters for a healthy and KOA subject is a limitation as it can alter the tissue mechanics significantly. In future work, experimental non-invasive characterization of subject-specific material parameters (e.g., using Raman spectroscopy or MRI) may allow more fine-tuning of the estimation of mechanical parameters driving the mechanoregulatory algorithm. However, in this study, given the consistent implementation of the entire modeling workflow in both subjects and the inclusion of subjects with similar demographics, we are confident in the comparability of the FE model results and the validity of the conclusions regarding the relative differences between the control and KOA subjects. Furthermore, Figure 3, demonstrates that the strains in the KOA subject are higher than those in the control subject for the vast majority of the stance phase indicating a noticeable disparity between the FE model results of the two subjects. As a next step, an additional uncertainty analysis and validation, particularly in a larger cohort of subjects would need to be performed to ensure the accurate prediction of the knee joint’s mechanical environment for each individual.

Altered cartilage tissue response, namely, local strain concentrations, seem to be present at the origin of the collagen split lines where there is a discontinuity in the fibrils. This could be an underlying cause of volume surpassing the MSS threshold in the control subject, suggesting PG depletion. These local strain concentrations can be considered and compensated for in adaptive modeling workflows. Studies by Párraga Quiroga et al. (2017) and Seyed and Makris. (2020) have proposed a non-localization theory, where strains are averaged over a certain volume. This would change the interpretation of these results regarding PG depletion in adaptive modeling workflows.

Additionally, there is an ambiguity regarding the exact thresholds of degeneration, with different studies reporting alternative thresholds as high as 50% for the MSS (Orozco et al., 2018; Orozco et al., 2021). In future studies, analysis of SFD and MSS during gait in a larger cohort of KOA patients presenting with and without disease progression in terms of structural cartilage degeneration will allow further refining of the threshold values of these functional biomarkers allowing identification of patients at risk of tissue degeneration. Furthermore, it should be noted that the relevance of the defined thresholds of the mechanoregulatory factors (MSS and SFD) for *in vivo* conditions is not clear, given that the current thresholds were selected based on outcomes from *in vitro* experiments (Eskelinen et al., 2019; Elahi et al., 2021). Further basic research should warrant the development of new technology to determine these thresholds *in vivo* and also to allow further finetuning, e.g., based on disease-specific mechanisms.

The focus of this study was to compare the effects of subject-specific loading and boundary conditions on the predicted mechanical environment of cartilage and meniscus in a healthy and early-stage KOA subject. In future studies, a sensitivity analysis can be conducted to specifically evaluate the influence of various subject-specific loading and boundary conditions or a combination of these conditions, which serve as an input to the FE model (such as TF anterior-posterior translation, TF medial-lateral translation, TF internal-external rotation, knee flexion angle, varus-valgus moment, and superior-inferior knee joint contact force), on the resulting tissue strains.

## 5 Conclusion

Patient-specific gait data at baseline were processed through a multi-scale *in silico* modeling approach to analyze key mechanoregulatory factors for cartilage degeneration. These mechanoregulatory factors were able to distinguish between a healthy and progressive early-stage KOA subject and even more, able to identify the tibial compartment at risk of progression. Furthermore, evaluated tissue responses in the menisci can be related to specific OA loading conditions. Therefore, the mechanoregulatory factors analyzed could potentially serve as functional biomarkers allowing discrimination of patients at risk of (fast) disease progression. As such, this study presents a first step towards in silico-informed screening in the context of musculoskeletal disorders. Following further validation in larger cohorts, this could not only aid in earlier diagnosis, personalized treatment provision and prediction of progression as well as monitoring for patients with KOA, but may also provide novel fundamental insights into the intricate interplay between mechanical tissue loading during locomotion, the local cartilage micro-environment *in vivo* and consequent cartilage tissue degeneration.

## Data Availability

The data analyzed in this study is subject to the following licenses/restrictions: The data was originally collected for other studies. Requests to access these datasets should be directed to IJ, ilse.jonkers@kuleuven.be.
