# Supplementary material for: Signatures of disease progression in knee osteoarthritis: insights from an integrated multi-scale modeling approach, a proof of concept
Source: Front Bioeng Biotechnol. 2023 Jul 27;11:1214693. doi: 10.3389/fbioe.2023.1214693 (PMC10413555; doi:10.3389/fbioe.2023.1214693)
Supplement: Supplementary file 1 [file DataSheet1.docx]

Supplementary Material

Signatures of disease progression in knee osteoarthritis: insights from an integrated multi-scale modeling approach

Ikram Mohout*, Seyed Ali Elahi, Amir Esrafilian, Bryce A. Killen, Rami K. Korhonen, Sabine Verschueren, Ilse Jonkers

*** Correspondence:** Ikram Mohout: ikram.mohout@kuleuven.be

# Subject-specific loading and boundary conditions

Figure S1 shows the post-processed patient specific loading and boundary conditions in the 6 degrees of freedom that were used to drive the finite element model. The raw data extracted from the musculoskeletal workflow was filtered using a second order zero-lag Butterworth low-pass filter with a 6Hz cut-off frequency and smoothed.


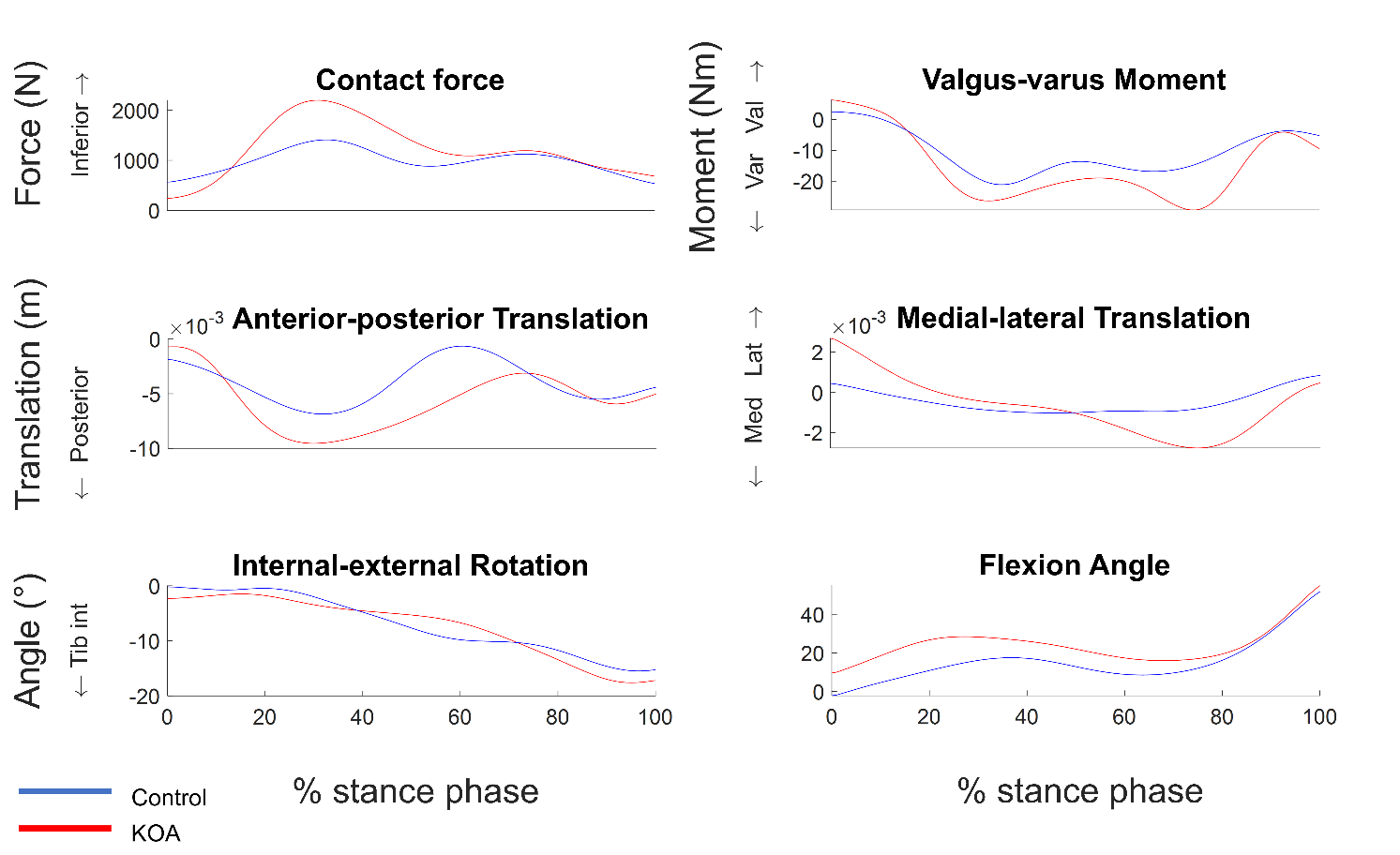


Figure S1 Patient specific boundary conditions in the 6 degrees of freedom extracted from the musculoskeletal model: Contact force inferior superior direction (N), Valgus-varus Moment (Nm), Anterior-posterior Translation (m), Medial-lateral Translation (m), internal-external rotation (°), flexion angle (°).

# Material properties of the FE model

## Cartilage and menisci

Cartilage was modelled as fibril reinforced poroviscoelastic (FRPVE) material (Wilson *et al.*, 2004; Eskelinen *et al.*, 2019). The 3D collagen network consisted of 17 fibrils. More specifically, 4 primary fibrils that run from the subchondral bone towards the superficial layer of the cartilage and split up following the arcade model of Benninghoff (1925) and 13 randomized secondary fibrils (Wilson *et al.*, 2004). Figure S2 illustrates the collagen fibril orientation in a depth wise manner as well as at the superficial layer of the cartilage tissue.


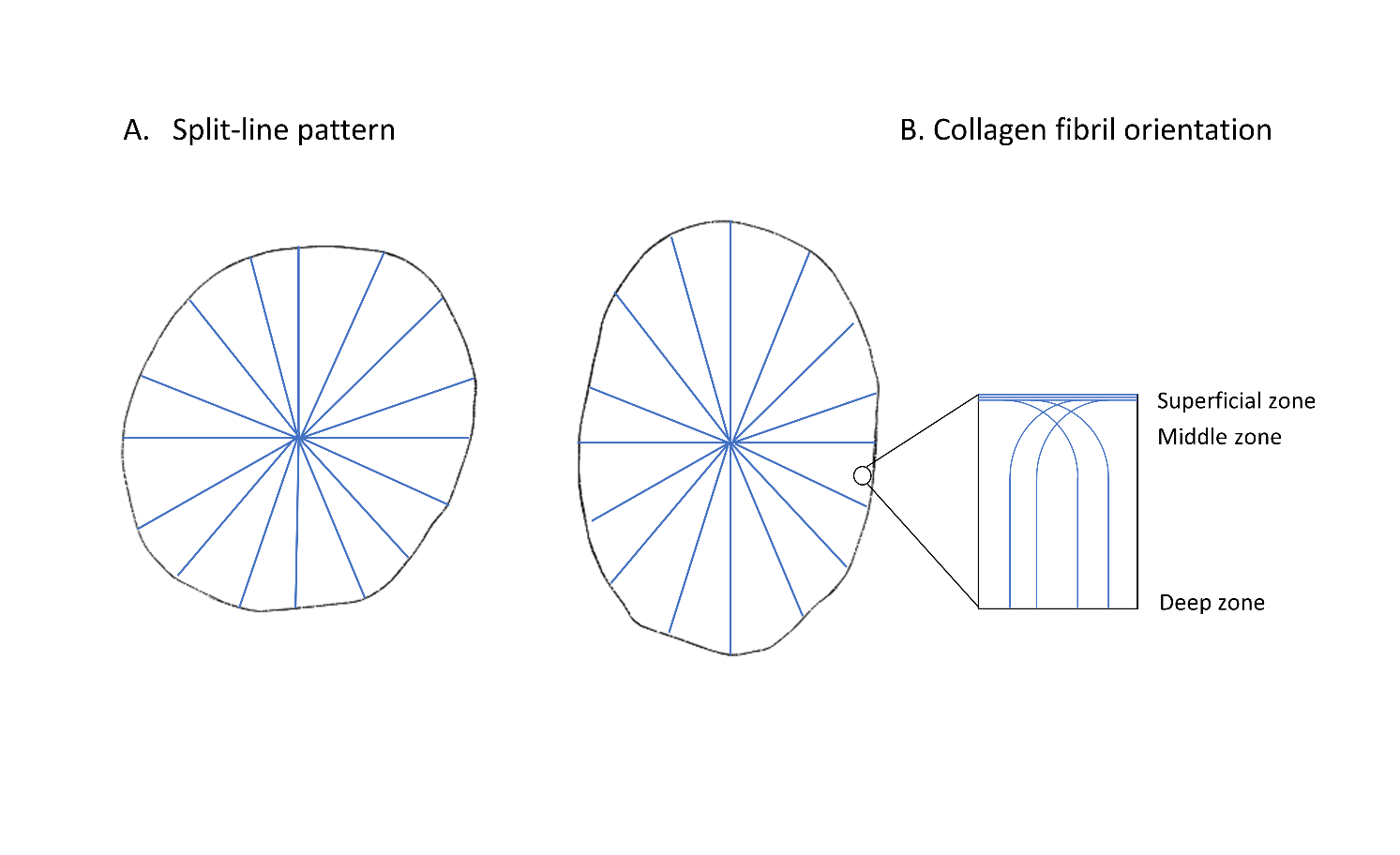

**Figure S2 Illustration of (A) the superficial collagen fibril orientation of the tibial cartilage according to the split-line patterns and (B) orientation of the collagen fibrils through the depth of the tibial cartilage.**

These fibrils exhibited viscoelastic properties with stress-strain behavior:

| $\sigma_{f}=\left\{ \begin{matrix} -\frac{\eta}{2\sqrt{\left( \boldsymbol{\sigma}_{\boldsymbol{f}}-E_{0}\boldsymbol{\varepsilon}_{\boldsymbol{f}} \right)E_{\varepsilon}}}\dot{\boldsymbol{\sigma}_{\boldsymbol{f}}}+E_{0}\boldsymbol{\varepsilon}_{\boldsymbol{f}}+\left( \eta+\frac{\eta E_{0}}{2\sqrt{\left( \boldsymbol{\sigma}_{\boldsymbol{f}}-E_{0}\boldsymbol{\varepsilon}_{\boldsymbol{f}} \right)E_{\varepsilon}}} \right)\dot{\boldsymbol{\varepsilon}_{\boldsymbol{f}}}, \varepsilon_{f}\geq0 \\ 0, \varepsilon_{f}<0 \end{matrix} \right.$, | (S1) |
| --- | --- |

where $\boldsymbol{\sigma}_{\boldsymbol{f}}$ and $\boldsymbol{\varepsilon}_{\boldsymbol{f}}$ are the fibril stress and logarithmic strain and $\dot{\boldsymbol{\sigma}_{\boldsymbol{f}}}$ and $\dot{\boldsymbol{\varepsilon}_{\boldsymbol{f}}}$ are the stress and strain rates, respectively. η is the damping coefficient, E_0_ is initial modulus and E_ɛ_ is the strain dependent modulus of the fibril network. The stress tensor formulated with respect to the primary and secondary collagen fibrils are the following:

| $\begin{matrix} \boldsymbol{\sigma}_{\boldsymbol{f,p}}=C\boldsymbol{\sigma}_{\boldsymbol{f}} \\ \boldsymbol{\sigma}_{\boldsymbol{f,s}}=\boldsymbol{\sigma}_{\boldsymbol{f}} \end{matrix}$, | (S2) |
| --- | --- |

where $\boldsymbol{\sigma}_{\boldsymbol{f,p}}$ and $\boldsymbol{\sigma}_{\boldsymbol{f,s}}$ are the stress tensors of the primary and secondary fibrils respectively and C is the ratio between the primary and secondary fibrils.

A compressive neo-Hookean material model was used to model the non-fibrillar matrix reflecting the PG content. The matrix stress is as followed:

| $\boldsymbol{\sigma}_{\mathrm{nf}}=\frac{1}{2}K_{\mathrm{nf}}\left( J\boldsymbol{-}\frac{1}{J} \right)\mathbf{I}+\frac{G_{\mathrm{nf}}}{J}\left( \mathbf{F}\mathbf{F}^{T}\boldsymbol{-}J^{\frac{\boldsymbol{2}}{\boldsymbol{3}}}\mathbf{I} \right)$, | (S3) |
| --- | --- |

where **I** is the identity tensor, **F** is the deformation gradient and J is the determinant of **I**. K_m_ and G_m_ are the bulk and shear moduli which were defined as:

| $K_{m}=\frac{E_{m}}{3\left( {1-2\nu}_{m} \right)}$ | (S4) |
| --- | --- |

| $G_{m}=\frac{E_{m}}{2\left( {1+\nu}_{m} \right)}$, | (S5) |
| --- | --- |

where E_m_ is the Young’s modulus and ν_m_ is the Poisson’s ratio of the non-fibrillar matrix. Thus, the total cartilage stress is:

| $\boldsymbol{\sigma}_{tot}=\boldsymbol{\sigma}_{nf}+ \sum_{i=1}^{totalf} {\boldsymbol{\sigma}_{f}}^{i}-p\boldsymbol{I}$, | (S6) |
| --- | --- |

where p is the fluid pressure and totalf is the total number of fibrils, namely 17 (4 primary and 13 secondary).

The fluid flow in the cartilage tissue was defined based on Darcy’s law:

| $\boldsymbol{q}=-k\nabla p$, | (S7) |
| --- | --- |

where **q** is the vector of the fluid flow flux, $\nabla p$ pore fluid pressure gradient and k is the permeability. The permeability is deformation dependent and was defined as (van der Voet, 1997):

| $k=k_{0}\left( \frac{1+e}{1+e_{0}} \right)^{M}$, | (S8) |
| --- | --- |

where k_0_ is the initial permeability, e_0_ and e are the initial and current void ratio and M is a constant. The depth-dependent fluid fraction was defined as:

| $n_{f,eq}=0.80-0.15h_{z}$, | (S9) |
| --- | --- |

where h_z_ is the normalized depth, defined as 0 at the surface and 1 at the bone interface of the cartilage.

For the menisci a fibril reinforced poroelastic (FRPE) material model was implemented. This model is analogous to the FRPVE model, apart from collagen fibrils where the stress-strain behavior was defined as:

| $\sigma_{f,menisci}=\left\{ \begin{matrix} E_{0}\boldsymbol{\varepsilon}_{\boldsymbol{f}}\boldsymbol{+}\frac{\boldsymbol{1}}{\boldsymbol{2}}E_{ɛ}{\boldsymbol{\varepsilon}_{\boldsymbol{f}}}^{\boldsymbol{2}}, \varepsilon_{f}\geq0 \\ 0, \varepsilon_{f}<0 \end{matrix} \right.$, | (S10) |
| --- | --- |

where $\sigma_{f,menisci}$ is the stress in the collagen fibrils of the menisci.

In addition, the fluid fraction ($n_{f,eq})$of the menisci is not depth-dependent (0.72). The meniscal horns are modelled as linear springs with ~30 springs per horn. The total spring constant of each meniscal horn is 350 N/mm (Villegas *et al.*, 2007).

Table 1 Material parameters of cartilage and menisci (Wilson *et al.*, 2004; Julkunen *et al.*, 2007; Makris, Hadidi and Athanasiou, 2011; Dabiri and Li, 2013) :

| Geometry | E_m_ (MPa) | E_0_ (MPa) | E_ɛ_ (MPa) | ν_m_ (-) | k_0_  (mm^4^/Ns) | M (-) | η (MPa s) | C (-) |
| --- | --- | --- | --- | --- | --- | --- | --- | --- |
| Femoral cartilage | 0.215 | 0.92 | 150 | 0.15 | 6x10^-3^ | 5.09 | 1062 | 12.16 |
| Tibial cartilage | 0.106 | 0.18 | 23.6 | 0.15 | 18x10^-3^ | 15.64 | 1062 | 12.16 |
| Menisci | 0.500 | 28 | - | 0.36 | 1.25x10^-3^ | 12.16 | - | 12.16 |

## Ligaments

The ligaments were modelled as nonlinear spring bundles, the stiffness was 9840N for the anterior cruciate ligament (ACL), 6000 N for the posterior cruciate ligament (PCL), 2400N for the lateral collateral ligament (LCL) and 8000 N for the medial collateral ligament (MCL) (Lenhart *et al.*, 2015). The force-strain relationship is defined as (Blankevoort and Huiskes, 1991):

| $f=\left\{ \begin{aligned} 0, ɛ<0 \\ \frac{1}{2}K\frac{ɛ^{2}}{ɛ_{l}} 0 \leqɛ\leq{2ɛ}_{l} \\ K(ɛ-ɛ_{l}), ɛ< 2ɛ_{l} \end{aligned} \right.$, | (S11) |
| --- | --- |

where f is the tensile force, K is stiffness of the ligaments and $ɛ_{l}$ is the end of the toe region (taken as 0.03) and $ɛ$ is the current ligament strain.

## Bone

Part of the tibia bone was modeled for the purpose of facilitating the convergence of the model to a numerical solution. Tibia bone was modeled as linear elastic material with a Young’s modulus of 80GPa which reflects the Young’s modulus of bone apatite (Currey, 2004).

# References

Benninghoff, A. (1925) ‘Form und Bau der Gelenkknorpel in ihren Beziehungen zur Funktion - Erste Mitteilung: Die modellierenden und formerhaltenden Faktoren des Knorpelreliefs’, *Zeitschrift für Anatomie und Entwicklungsgeschichte*, 76(1–3), pp. 43–63. doi: 10.1007/BF02134417.

Blankevoort, L. and Huiskes, R. (1991) ‘Ligament-bone interaction in a three-dimensional model of the knee’, *Journal of Biomechanical Engineering*, 113(3), pp. 263–269. doi: 10.1115/1.2894883.

Currey, J. (2004) ‘Incompatible mechanical properties in compact bone’, *Journal of Theoretical Biology*, 231(4), pp. 569–580. doi: 10.1016/j.jtbi.2004.07.013.

Dabiri, Y. and Li, L. P. (2013) ‘Influences of the depth-dependent material inhomogeneity of articular cartilage on the fluid pressurization in the human knee’, *Medical Engineering & Physics*, 35(11), pp. 1591–1598. doi: 10.1016/J.MEDENGPHY.2013.05.005.

Eskelinen, A. S. A. *et al.* (2019) ‘Maximum shear strain-based algorithm can predict proteoglycan loss in damaged articular cartilage’, *Biomechanics and Modeling in Mechanobiology*, 18(3), pp. 753–778. doi: 10.1007/s10237-018-01113-1.

Julkunen, P. *et al.* (2007) ‘Characterization of articular cartilage by combining microscopic analysis with a fibril-reinforced finite-element model’, *Journal of Biomechanics*, 40(8), pp. 1862–1870. doi: 10.1016/j.jbiomech.2006.07.026.

Lenhart, R. L. *et al.* (2015) ‘Prediction and Validation of Load-Dependent Behavior of the Tibiofemoral and Patellofemoral Joints During Movement’, *Annals of Biomedical Engineering*, 43(11), pp. 2675–2685. doi: 10.1007/s10439-015-1326-3.

Makris, E. A., Hadidi, P. and Athanasiou, K. A. (2011) ‘The knee meniscus: Structure-function, pathophysiology, current repair techniques, and prospects for regeneration’, *Biomaterials*, 32(30), pp. 7411–7431. doi: 10.1016/j.biomaterials.2011.06.037.

Villegas, D. F. *et al.* (2007) ‘Failure properties and strain distribution analysis of meniscal attachments’, *Journal of Biomechanics*, 40(12), pp. 2655–2662. doi: 10.1016/j.jbiomech.2007.01.015.

van der Voet, A. (1997) ‘A comparison of finite element codes for the solution of biphasic poroelastic problems.’, *Proceedings of the Institution of Mechanical Engineers. Part H, Journal of engineering in medicine*, pp. 209–211. Available at: https://pubmed.ncbi.nlm.nih.gov/9184461/ (Accessed: 21 April 2023).

Wilson, W. *et al.* (2004) ‘Stresses in the local collagen network of articular cartilage: A poroviscoelastic fibril-reinforced finite element study’, *Journal of Biomechanics*, 37(3), pp. 357–366. doi: 10.1016/S0021-9290(03)00267-7.
